# Supplementary material for: Harbour porpoise responses to pile-driving diminish over time
Source: R Soc Open Sci. 2019 Jun 26;6(6):190335. doi: 10.1098/rsos.190335 (PMC6599776; doi:10.1098/rsos.190335)
Supplement: Supplementary Methods Tables Figures & R code for Graham et al Porpoise responses to piling [file rsos190335supp1.docx]

**Details of underwater noise modelling**

*Source model*

The source level estimate for pile driving was calculated using an energy conversion model (De Jong and Ainslie, 2008), whereby a proportion of the hammer energy is converted to acoustic energy:

| $\boldsymbol{SL}_{\boldsymbol{E}}\boldsymbol{=120+10}\boldsymbol{log}_{\boldsymbol{10}}\left( \frac{\boldsymbol{\beta Ec}_{\boldsymbol{0}}\boldsymbol{\rho}}{\boldsymbol{4}\boldsymbol{\pi}} \right)$ | (1) |  |  |  |  |
| --- | --- | --- | --- | --- | --- |

where $E$ is the hammer energy in joules, ${SL}_{E}$ is the source level energy for a single strike at hammer energy $E$, $\boldsymbol{\beta}$ is the acoustic energy conversion efficiency, $c_{0}$ is the speed of sound in seawater in m s^-1^, and $\rho$ is the density of seawater in kg m^-3^.

This yields an estimate of the source level in units of sound exposure level (dB re 1 µPa^2^ s). This energy is then distributed across the frequency spectrum based on previous measurements of impact piling (Ainslie *et al.*, 2012).

Equation 1 was used to compute the source level energies, using an acoustic energy conversion efficiency of 0.5%, which assumes that 0.5% of the hammer energy is converted into acoustic energy. This energy conversion factor is in keeping with current understanding of how much hammer energy is converted to noise (Dahl and Reinhall, 2013; Zampolli *et al.*, 2013; Dahl *et al.*, 2015). The source level energy from Equation 1 can then be expressed as a single-strike SEL (SEL_ss_) for the corresponding hammer energy.

*Propagation model*

At frequencies at or below 1.25 kHz, a parabolic equation propagation loss model was used (RAM; Collins, 1993). This model becomes computationally inefficient at higher frequencies (Farcas et al, 2016), and so an energy flux model was used (Weston, 1971) at frequencies >1.25 kHz. The model was implemented using custom-written codes in Python. Sound levels were computed in one-third octave frequency bands, based on 8 separate frequencies computed within each band.

The sound speed profile was computed from contemporaneous water column parameters (temperature, salinity) extracted from Copernicus^[[1]](#footnote-1)^. The seabed properties modelled were 40 m of sand above a hard basement, based on cross-sectional data of the Moray Firth from the British Geological Survey. Bathymetry data for the site was provided by the wind farm developer at 90 m resolution.

*Corroboration with measurements*

To assess the consistency of the modelled predictions with the measured data, data for a subset of seven pile strikes measured simultaneously at three recording locations was compared to modelled predictions. The recorders were located at 2.0 km, 7.6 km, and 10.2 km from the source. The pile strikes were selected for high signal-to-noise ratio at all three measurement locations, and had hammer energies of 508-716 kJ, toward the upper end of the hammer energy range used in the operation (from 167 kJ up to 1048 kJ for some piles). The measured SEL_ss_ was computed for each of the 21 strikes for comparison against the modelled predictions of SEL_ss_.

Three metrics were computed, consistent with the dose metrics used in the analysis of porpoise responses: unweighted SEL_ss_, NOAA-weighted SEL_ss_ for high-frequency cetaceans, and audiogram-weighted SELss based on Kastelein et al (2010); see Section 2 of main paper. The audiogram weighting was extrapolated at frequencies below 250 Hz as shown in Figure S1.


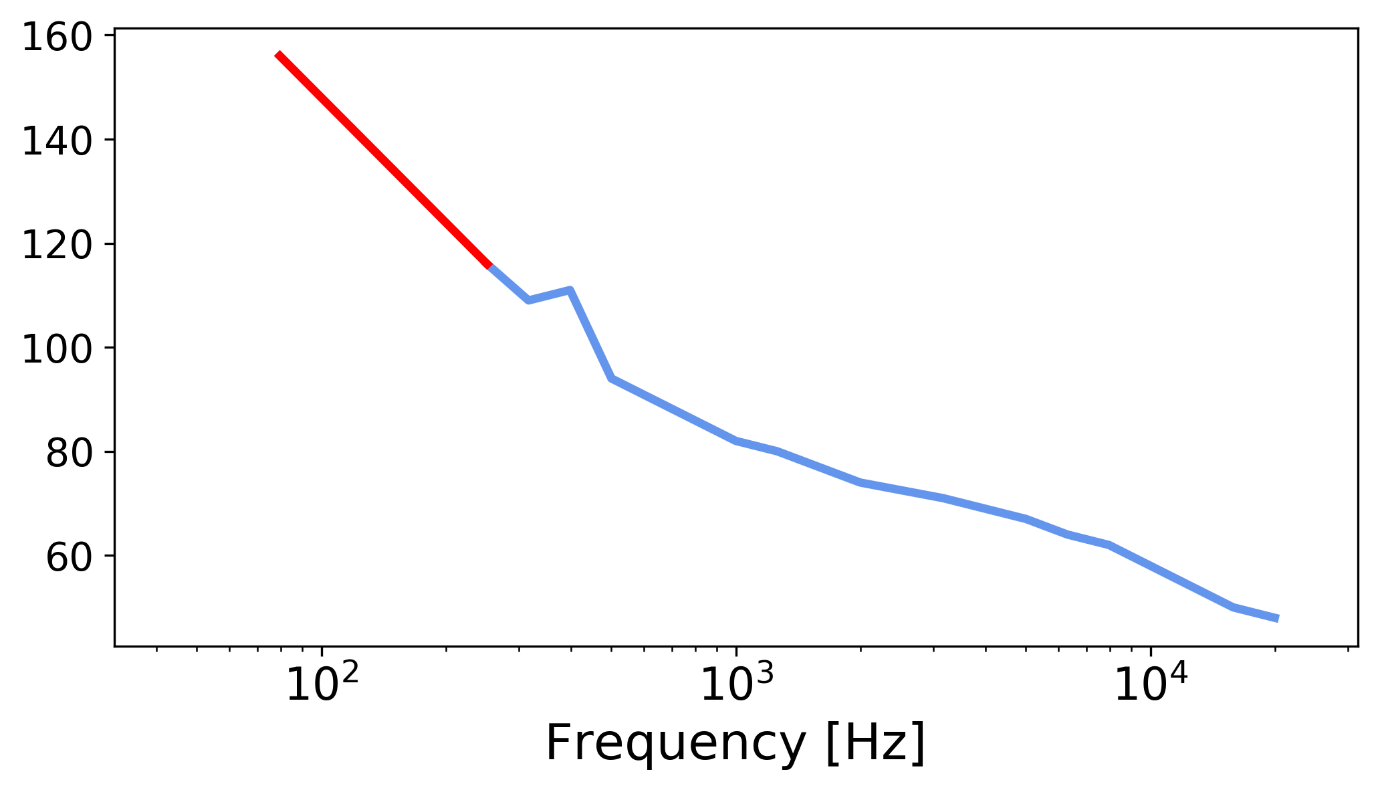


Figure S1. Audiogram used for computation of harbour porpoise audiogram-weighted received sound levels. Blue line shows audiogram data reported in Kastelein et al (2010). Red line is extrapolation used to cover frequencies <250 Hz, which were not measured in the Kastelein study.

The errors in the model were lower at greater ranges (Table S1), where harbour porpoise responses were expected to occur. RMS errors were within 3 dB for the further two locations, and substantially higher at 2 km (up to 6.6 dB). However, optimising the propagation model further to improve the short-range accuracy would compromise the accuracy at longer range, which was considered more significant for the predictions of harbour porpoise response.

Table S1. RMS errors for model predictions of SEL_ss_ compared to field measurements at three sites.

| Location ID | Range (km) | RMS error (dB) in predictions of SEL_ss_ | | |
| --- | --- | --- | --- | --- |
|  |  | Unweighted | NOAA-weighted | Audiogram weighted |
| 738 | 2.0 | 6.58 | 5.45 | 6.50 |
| 753 | 7.6 | 1.95 | 1.69 | 2.65 |
| 727 | 10.2 | 0.80 | 1.44 | 2.89 |

**References**

Ainslie, M.A., de Jong, C.A.F., Robinson, S.P. & Lepper, P.A. (2012). What is the source level of pile-driving noise in water? *In*: Eff. Noise Aquat. Life (eds. Popper, A.N. & Hawkins, A.D.). Springer, NY, pp. 445–448.

Collins, M.D. (1993). A split-step Padé solution for the parabolic equation method. J. Acoust. Soc. Am., 93, 1736–1742.

Dahl, P.H., de Jong, C.A.F. & Popper, A.N. (2015). The Underwater Sound Field from Impact Pile Driving and Its Potential Effects on Marine Life. Acoust. Today, 11.

Dahl, P.H. & Reinhall, P.G. (2013). Beam forming of the underwater sound field from impact pile driving. J. Acoust. Soc. Am., 134, EL1-6.

Farcas, A., Thompson, P.M. & Merchant, N.D. (2016). Underwater noise modelling for environmental impact assessment. Environ. Impact Assess. Rev., 57, 114–122.

De Jong, C.A. f & Ainslie, M.A. (2008). Underwater radiated noise due to the piling for the Q7 Offshore Wind Park. J. Acoust. Soc. Am., 123, 2987.

Kastelein, R. A., Hoek, L., de Jong, C. A. F., and Wensveen, P. J. 2010. The effect of signal duration on the underwater detection thresholds of a harbor porpoise (Phocoena phocoena) for single frequency-modulated tonal signals between 0.25 and 160 kHz. The Journal of the Acoustical Society of America, 128, 3211–3222.

Weston, D. E. 1971. Intensity-range relations in oceanographic acoustics. Journal of Sound and Vibration, 18: 271–287.

Zampolli, M., Nijhof, M.J.J., de Jong, C.A.F., Ainslie, M.A., Jansen, E.H.W. & Quesson, B.A.J. (2013). Validation of finite element computations for the quantitative prediction of underwater noise from impact pile driving. J. Acoust. Soc. Am., 133, 72–81.

Table S2. Summary of piling activity at the 17 OTM/turbine locations used in the analysis from 01/04/2017 – 02/12/2017 at the BOWL construction site.

| Turbine | Start time | End time | Piling duration (hours) | Total blow count | Max hammer energy | Interval (days) | Piling order | Latitude | Longitude | ADD used |
| --- | --- | --- | --- | --- | --- | --- | --- | --- | --- | --- |
| G7 | 02/04/2017 06:51 | 02/04/2017 18:20 | 7.10 | 9281 | 662 | NA | 1 | 58.25007 | -2.88057 | Y |
| F8 | 07/04/2017 17:52 | 08/04/2017 01:35 | 5.78 | 10196 | 951 | 5.0 | 2 | 58.25685 | -2.89583 | Y |
| E2 | 14/04/2017 02:38 | 14/04/2017 11:49 | 4.67 | 7741 | 861 | 4.2 | 4 | 58.19116 | -2.93547 | Y |
| J5 | 04/05/2017 06:36 | 04/05/2017 15:45 | 7.05 | 10877 | 737 | 13.7 | 8 | 58.23653 | -2.85008 | Y |
| G5 | 10/05/2017 07:34 | 10/05/2017 15:39 | 5.74 | 10922 | 958 | 4.3 | 10 | 58.22937 | -2.88757 | Y |
| F5 | 17/05/2017 01:22 | 17/05/2017 07:34 | 4.64 | 6938 | 884 | 5.2 | 12 | 58.2258 | -2.90629 | Y |
| J8 | 11/06/2017 11:20 | 11/06/2017 15:25 | 3.27 | 6292 | 1209 | 8.1 | 23 | 58.26758 | -2.83955 | Y |
| E4 | 01/07/2017 13:13 | 02/07/2017 05:17 | 7.96 | 16127 | 1408 | 9.0 | 32 | 58.21186 | -2.92851 | Y |
| L8 | 24/07/2017 14:52 | 24/07/2017 22:38 | 5.48 | 11195 | 1765 | 6.0 | 47 | 58.27471 | -2.80203 | Y |
| G12 | 18/08/2017 12:13 | 18/08/2017 21:02 | 3.52 | 6939 | 1029 | 4.5 | 61 | 58.30181 | -2.86306 | N |
| F11 | 07/09/2017 03:27 | 07/09/2017 10:05 | 4.47 | 8399 | 1356 | 5.5 | 70 | 58.2879 | -2.88534 | Y |
| E11 | 18/09/2017 15:56 | 19/09/2017 01:07 | 6.18 | 13578 | 883 | 4.4 | 75 | 58.28431 | -2.90411 | Y |
| H13 | 07/10/2017 02:48 | 07/10/2017 08:33 | 3.44 | 6169 | 1045 | 15.2 | 79 | 58.31574 | -2.84077 | Y |
| H10 | 26/10/2017 18:34 | 27/10/2017 02:01 | 4.62 | 9042 | 753 | 16.5 | 83 | 58.2847 | -2.85131 | Y |
| G9 | 04/11/2017 05:20 | 04/11/2017 12:51 | 5.29 | 10817 | 864 | 8.2 | 84 | 58.27078 | -2.87358 | Y |
| H9 | 14/11/2017 20:41 | 15/11/2017 03:07 | 4.01 | 8141 | 657 | 10.3 | 85 | 58.27435 | -2.85482 | Y |
| J10 | 02/12/2017 16:54 | 02/12/2017 23:39 | 4.45 | 8290 | 1278 | 17.6 | 86 | 58.28827 | -2.83254 | Y |

Table S3. Models of harbour porpoise behavioural responses to piling. Response was defined as a proportional decrease in harbour porpoise occurrence > 0.5 in the 24 or 12 hours after cessation of piling. Relationships were modelled using generalised linear mixed models with a binomial error distribution and the probit link function. Distance from piling, received single-pulse sound exposure levels (SEL) with different weighting functions, ADD use and vessel activity were used as explanatory variables. All models included a random effect of CPOD sampling site combined with CPOD identity.

| Response Length | Number of Piling Locations | Sample Size | CPODs used | Model | AIC | Marginal R^2^ |
| --- | --- | --- | --- | --- | --- | --- |
| 24-hour | 17 | 654 | All | **Log(distance)*piling order + no. vessel locations_1km** | **619.4** | **0.15** |
|  |  |  |  | **Audiogram SS_SEL *piling order + no. vessel locations_1km** | **621.0** | **0.14** |
|  |  |  |  | Log(distance)*piling order + vessel presence_500m | 619.3 | 0.13 |
|  |  |  |  | NOAA SS_SEL *piling order + no. vessel locations_1km | 623.0 | 0.14 |
|  |  |  |  | HF cetacean SS_SEL *piling order + no. vessel locations_1km | 624.8 | 0.14 |
|  |  |  |  | Unweighted SS_SEL *piling order + no. vessel locations_1km | 627.0 | 0.14 |
|  | 18 (includes 2^nd^ location without ADD mitigation) | 700 | All | Log(distance)*piling order + no. vessel locations_1km | 656.6 | 0.15 |
|  |  |  |  | Audiogram SS_SEL *piling order + no. vessel locations_1km | 658.1 | 0.15 |
|  | 17 | 641 | > 1km (from piling) | Log(distance)*piling order + no. vessel locations_1km | 606.8 | 0.13 |
|  |  |  |  | Audiogram SS_SEL *piling order + no. vessel locations_1km | 608.7 | 0.13 |
| 12-hour | 17 | 623 | All | **Log(distance)*piling order + ADD + no. vessel locations_500m** | **653.4** | **0.22** |
|  |  |  |  | Log(distance)*piling order + ADD + no. vessel locations_500m + piling duration | 653.5 | 0.22 |
|  |  |  |  | **Audiogram SS_SEL *piling order + ADD + no. vessel locations_500m** | **657.3** | **0.23** |
|  |  |  |  | NOAA SS_SEL *piling order + ADD + no. vessel locations_500m | 658.0 | 0.24 |
|  |  |  |  | HF cetacean SS_SEL *piling order + ADD + no. vessel locations_500m | 659.9 | 0.24 |
|  |  |  |  | Unweighted SS_SEL *piling order + ADD + no. vessel locations_500m | 660.6 | 0.25 |
|  | 18 (includes 2^nd^ location without ADD mitigation) | 669 | All | Log(distance)*piling order + ADD + no. vessel locations_500m | 683.9 | 0.26 |
|  |  |  |  | Audiogram SS_SEL *piling order + ADD + no. vessel locations_500m | 687.3 | 0.27 |
|  | 17 | 612 | > 1km (from piling) | Log(distance)*piling order + ADD + no. vessel locations_500m | 637.0 | 0.29 |
|  |  |  |  | Audiogram SS_SEL *piling order + ADD + no. vessel locations_500m | 641.5 | 0.29 |

Table S4. Modelled relationship of harbour porpoise behavioural response to piling using subset of piling events (n = 9) preceded by at least a 192-hour break in piling. Response was defined as a proportional decrease in harbour porpoise occurrence > 0.5 in the 24 hours after cessation of piling. Relationship was modelled using generalised linear models with a binomial error distribution and the probit link function. Distance from piling, cumulative number of locations piled (piling order), ADD use and the number of AIS vessel locations within 1km were used as explanatory variables. The model does not include a random effect (of CPOD sampling site combined with CPOD identity) because with the reduced data set there were insufficient data to estimate a random effect of this complexity and its inclusion caused singularity issues.

| Model |  | Estimate | Std. error | *z* Value | *P* | AIC |
| --- | --- | --- | --- | --- | --- | --- |
| 24-h response ~ log(distance)*piling order + no. vessel locations_1km | | | | | | 322.1 |
|  | (Intercept) | 0.5488 | 0.1960 | 2.800 | 0.005 |  |
|  | Log(distance):piling order | 0.1932 | 0.0818 | 2.362 | 0.018 |  |
|  | Log(distance) | -0.4738 | 0.0768 | -6.168 | < 0.001 |  |
|  | Piling order | -0.6530 | 0.2053 | -3.180 | 0.001 |  |
|  | No. vessel locations_1km | 0.2004 | 0.1381 | 1.451 | 0.147 |  |
|  |  |  |  |  |  |  |


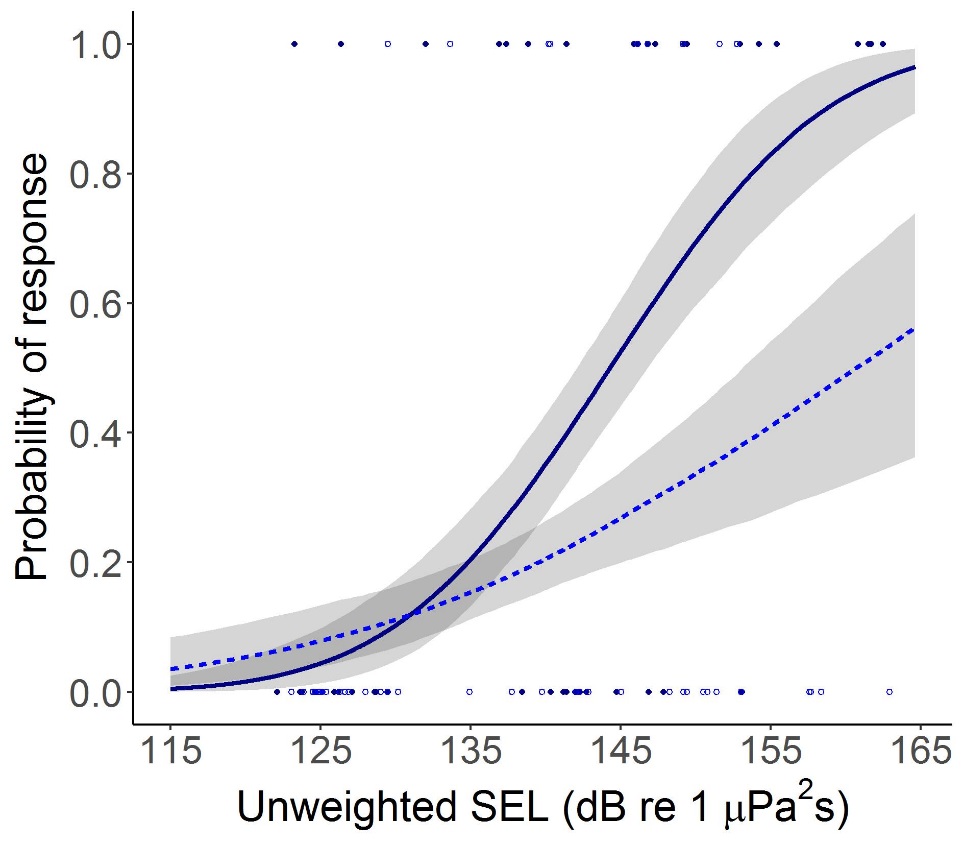


Figure S2. The probability of a harbour porpoise response (24h) in relation to the partial contribution of unweighted received single-pulse SEL for the first location piled (solid navy line) and the final location piled (dashed blue line), predicted assuming the number of AIS vessel locations within 1km = 0; confidence intervals (shaded areas) estimated for uncertainty in fixed effects only. Harbour porpoise occurrence was considered to have responded to piling when the proportional decrease in occurrence (DPH) exceeded a threshold of 0.5. Points show actual response data for the first location piled (filled navy circles) and the final location piled (open blue circles).

**R code to calculate the number of individuals displaced using behavioural-response curves.**

# using bootstrapped predictions for model of 24-h response with distance, piling order and number of AIS vessel locations within 1km

m8_24_preds <- read.csv("bootstrapped predictions m8_24.csv",header=T) # read in bootstrapped predictions

bands <- seq(0.5,26,0.5) # define 0.5 km bands up to 26 km

mid_bands <- seq(0.25,25.75,0.5) # define the mid-point of each 0.5 km band

disp_data <- data.frame(cbind(bands,mid_bands)) # create a data frame with the distance bands & mid-points

disp_data$fitprob_1st <- NA

disp_data$lciprob_1st <- NA

disp_data$uciprob_1st <- NA

m8_24_preds_1st <- m8_24_preds[m8_24_preds$zorder < -1,] # select the predictions for the 1^st^ piling location

for(i in 1:nrow(disp_data)){

temp <- m8_24_preds_1st[(m8_24_preds_1st$distance==disp_data$mid_bands[i]),] # select the predictions for the distance = the mid-point of the band

disp_data$fitprob_1st[i] <- temp$fit # save the fitted probability of observing a response

disp_data$lciprob_1st[i] <- temp$lci # save the lower 95% CI of the probability of observing a response

disp_data$uciprob_1st[i] <- temp$uci # save the upper 95% CI of the probability of observing a response

}

disp_data$fitprob_47th <- NA

disp_data$lciprob_47th <- NA

disp_data$uciprob_47th <- NA

m8_24_preds_47th <- m8_24_preds[(m8_24_preds$zorder > -1 & m8_24_preds$zorder < 0),] # select the predictions for the 47^th^ piling location

for(i in 1:nrow(disp_data)){

temp <- m8_24_preds_47th[(m8_24_preds_47th$distance==disp_data$mid_bands[i]),] # select the predictions for the distance = the mid-point of the band

disp_data$fitprob_47th[i] <- temp$fit # save the fitted probability of observing a response

disp_data$lciprob_47th[i] <- temp$lci # save the lower 95% CI of the probability of observing a response

disp_data$uciprob_47th[i] <- temp$uci # save the upper 95% CI of the probability of observing a response

}

disp_data$fitprob_86th <- NA

disp_data$lciprob_86th <- NA

disp_data$uciprob_86th <- NA

m8_24_preds_86th <- m8_24_preds[m8_24_preds$zorder > 0,] # select the predictions for the 86^th^ piling location

for(i in 1:nrow(disp_data)){

temp <- m8_24_preds_86th[(m8_24_preds_86th$distance==disp_data$mid_bands[i]),] # select the predictions for the distance = the mid-point of the band

disp_data$fitprob_86th[i] <- temp$fit # save the fitted probability of observing a response

disp_data$lciprob_86th[i] <- temp$lci # save the lower 95% CI of the probability of observing a response

disp_data$uciprob_86th[i] <- temp$uci # save the upper 95% CI of the probability of observing a response

}

disp_data$circle_area <- pi*((disp_data$bands)*(disp_data$bands)) # calculate the total area of each band

disp_data$band_area <- disp_data$circle_area[1]

# calculate the actual area of each band

disp_data$band_area[2:nrow(disp_data)] <- disp_data$circle_area[2:nrow(disp_data)]-disp_data$circle_area[1:(nrow(disp_data)-1)]

disp_data$no_inds <- disp_data$band_area*0.274 # assuming density of 0.274 per square km, calculate the number of individuals per band

porp_total <- sum(disp_data$no_inds) # 581.8984 # total number of individuals in area of 26 square kms

# calculate the number of individuals predicted to have responded in each band

disp_data$disp_fitinds_1st <- disp_data$no_inds*disp_data$fitprob_1st

disp_data$disp_lciinds_1st <- disp_data$no_inds*disp_data$lciprob_1st

disp_data$disp_uciinds_1st <- disp_data$no_inds*disp_data$uciprob_1st

disp_data$disp_fitinds_47th <- disp_data$no_inds*disp_data$fitprob_47th

disp_data$disp_lciinds_47th <- disp_data$no_inds*disp_data$lciprob_47th

disp_data$disp_uciinds_47th <- disp_data$no_inds*disp_data$uciprob_47th

disp_data$disp_fitinds_86th <- disp_data$no_inds*disp_data$fitprob_86th

disp_data$disp_lciinds_86th <- disp_data$no_inds*disp_data$lciprob_86th

disp_data$disp_uciinds_86th <- disp_data$no_inds*disp_data$uciprob_86th

sum(disp_data$disp_fitinds_1st) # 160.1016 - number of individuals displaced within 26 square kms at first piling location

(sum(disp_data$disp_fitinds_1st))/porp_total # 0.2751367 - proportion of individuals displaced within 26 square kms at first piling location

sum(disp_data$disp_lciinds_1st) # 120.4393 - lower 95% CI of number of individuals displaced within 26 square kms at first piling location

(sum(disp_data$disp_lciinds_1st))/porp_total # 0.2069766 - lower 95% CI of proportion of individuals displaced within 26 square kms at first piling location

sum(disp_data$disp_uciinds_1st) # 202.3612 - upper 95% CI of number of individuals displaced within 26 square kms at first piling location

(sum(disp_data$disp_uciinds_1st))/porp_total # 0.3477604 - upper 95% CI of proportion of individuals displaced within 26 square kms at first piling location

sum(disp_data$disp_fitinds_47th) # 127.8755

(sum(disp_data$disp_fitinds_47th))/porp_total # 0.2197558

sum(disp_data$disp_lciinds_47th) # 104.2591

(sum(disp_data$disp_lciinds_47th))/porp_total # 0.1791706

sum(disp_data$disp_uciinds_47th) # 151.6025

(sum(disp_data$disp_uciinds_47th))/porp_total # 0.2605308

sum(disp_data$disp_fitinds_86th) # 102.4047 - number of individuals displaced within 26 square kms at 86th piling location

(sum(disp_data$disp_fitinds_86th))/porp_total # 0.1759838 - proportion of individuals displaced within 26 square kms at 86th piling location

sum(disp_data$disp_lciinds_86th) # 74.76836 - lower 95% CI of number of individuals displaced within 26 square kms at 86th piling location

(sum(disp_data$disp_lciinds_86th))/porp_total # 0.1284904 - lower 95% CI of proportion of individuals displaced within 26 square kms at 86th piling location

sum(disp_data$disp_uciinds_86th) # 133.2839 - upper 95% CI of number of individuals displaced within 26 square kms at 86th piling location

(sum(disp_data$disp_uciinds_86th))/porp_total # 0.2290502 - upper 95% CI of proportion of individuals displaced within 26 square kms at 86th piling location

1. <http://marine.copernicus.eu/services-portfolio/access-to-products/> [↑](#footnote-ref-1)
